# Immunogenicity evaluation of altSonflex1-2–3 *Shigella* vaccine across mice, rats, and rabbits to inform human translational insights

**DOI:** 10.3389/fimmu.2025.1740821

**Published:** 2026-01-21

**Authors:** Valentina Caradonna, Renzo Alfini, Marika Pinto, Roberta Di Benedetto, Carlo Giannelli, Donata Medaglini, Elena Pettini, Miren Iturriza, Omar Rossi, Francesca Micoli, Francesca Mancini

**Affiliations:** 1 Laboratory of Molecular Microbiology and Biotechnology, Department of Medical Biotechnologies, University of Siena, Siena, Italy; 2 GlaxoSmithKline (GSK) Vaccines Institute for Global Health (GVGH), Siena, Italy

**Keywords:** altSonflex1-2-3, animal models, dose-response, GMMA, *Shigella*, vaccine

## Abstract

*Shigella*, a leading cause of bacillary dysentery, represents a significant global health challenge, particularly in low- and middle-income countries. Shigellosis predominantly affects children under the age of five and is associated with high morbidity and mortality rates. To address this burden, a generalized modules for membrane antigens (GMMA)-based vaccine, altSonflex1-2-3, incorporating *S. sonnei* and *S. flexneri* 1b, 2a, and 3a O-antigens, has been developed. This study aimed to evaluate and compare the immunogenicity of the altSonflex1-2–3 vaccine in mice, rats, and rabbits. Significant increase in O-antigen specific IgG response was observed in all animal models after one single injection, that further increased post-second vaccination in mice and rats for all antigens at all tested doses. In rabbits, booster effects were observed for all antigens, except for *S. flexneri* O-antigen at the highest dose and *S. sonnei* O-antigen at intermediate and high dose. This study showed how each species exhibited its own unique dose-response pattern against *S. flexneri* 1b and 3a. Instead, *S. flexneri* 2a consistently showed a positive dose-response in every model examined. A hook effect was observed for *S. sonnei* IgG across all models, with responses peaking at medium doses and decreasing at higher doses. This trend was most pronounced in mice and less evident in rats. Across all antigens, mice and rabbits exhibited more homogeneous immune responses to the 4 antigens, while rats showed numerically higher response to *S. sonnei* and *S. flexneri* 2a compared to *S. flexneri* 1b and 3a. Interestingly, this pattern in rats aligns more closely with responses recently observed in European adults. The vaccine has now advanced to Phase 2 clinical trials in the target population of 9-month-old infants, where different doses of the vaccine are being tested. Immune data collected will allow to further evaluate which preclinical model can better predict humoral response elicited in different age group populations. Expanding studies of this kind across different platforms and pathogens could provide valuable insights into the optimal animal models for supporting rapid vaccine design and development prior to clinical trials.

## 1 Introduction

*Shigella*, a genus of Gram-negative bacteria, is the causative agent of shigellosis, a severe form of dysentery that primarily affects children under five years in low- and middle- income countries (1). The disease is characterized by symptoms such as diarrhea, fever, and stomach cramps, and can lead to significant morbidity and mortality (2–5). Several studies conducted during the most recent years showed how the burden has been also complicated by the emergence of antimicrobial-resistant strains of *S. sonnei* and *S. flexneri*, causing outbreaks also in high-income countries where is diffused in populations like men who have sex with men (MSM) (6–12). For this reason, *Shigella* was inserted in the WHO Bacterial Priority Pathogens List (13). The development of effective vaccines against *Shigella* is a priority for public health organizations worldwide, with several candidates currently in clinical development (14).

One promising approach in the fight against *Shigella* involves the use of Generalized Modules for Membrane Antigens (GMMA). GMMA are outer membrane vesicles derived from genetically modified bacteria that present multiple antigens in their native conformation, potentially eliciting a robust immune response (15, 16). In the case of *Shigella*, GMMA are used as a delivery system for the O-antigen (OAg), a component of the lipopolysaccharide on the bacterial surface, which plays a crucial role in immune response against *Shigella* (17, 18). The OAg is also crucial because it defines the diversity within the *Shigella* genus, which is formed by four species and more than 50 serotypes distinguished based on the OAg structure (19, 20). altSonflex1-2–3 vaccine targets four *Shigella* serotypes (*S. sonnei*, *S. flexneri* 1b, 2a and 3a), aiming to provide broad protection against this diverse pathogen (21). Alhydrogel is used as adsorbent agent of the four GMMA and not as adjuvant, with the purpose of reducing potential GMMA reactogenicity (22, 23). The vaccine is currently undergoing Phase 2 clinical trials (NCT05073003 and NCT06663436) and resulted to be safe and immunogenic in European adults (NCT05073003) (24).

Animal models are essential for the evaluation of immune responses to vaccines besides the assessment of safety and efficacy (25, 26). Mice are commonly used in vaccine research due to their well-characterized immune system (25). Also, the ease of genetic manipulation of mice had led to the availability of numerous well characterized outbred strains which permits to evaluate variations in responses due to genetic differences (27). They are particularly useful for initial screening of vaccine candidates, studying the mechanisms of immune responses, and evaluating the efficacy of different adjuvants or comparing different platforms for vaccine design. Mice were selected due to their established use in preliminary immunogenic studies and the ability to give strong immune responses at very low doses that also correspond with their smaller body size and weight (28, 29). In addition to being cost-effective to maintain, mice offer benefits from an animal welfare perspective, supported by long-standing ethical guidelines developed over years of biomedical use. Moreover, among species with a fully functional immune system, mice are comparatively less neurologically developed. This species can also rapidly reach the age stage that is needed for immunological studies (young adults), making them a convenient species for studies that require rapid generation of data. However, mouse strains typically exhibit lower levels of complement compared to humans or to other rodents like rats (30), which can limit their predictive value for human immune responses. Rats offer certain advantages such as their larger size which allows for easier handling and sample collection. More importantly, activated T cells in humans and rats express major histocompatibility complex class II, and CD8 and CD4 expression is observed on macrophages in these species, a feature not shared by mice (31). Rats are used to study the immunogenicity, safety, and pharmacokinetics of vaccine candidates and therapeutics (32), providing valuable data that can help bridging the gap between preclinical findings and human clinical responses. Rats were utilized for the presence of a complex immune system, taking into account their intermediate body weight to ensure precise dosing (31). Finally, rabbits have been used for immunogenicity studies since they possess a sophisticated adaptive immune system with genes more similar to humans than those of rodents (33–35). Rabbit antibody repertoire, characterized by high affinity and specificity, has been instrumental in generating both polyclonal and monoclonal antibodies for diagnostic and therapeutic applications (36, 37). Moreover, rabbits can typically receive the full human dose of a vaccine (38, 39), and their body weight is relatively similar to that of infants, unlike other species where lower doses must be administered, complicating allometric calculations (40). Rabbit was the model that was chosen for providing safety and toxicology data on GMMA-based vaccines against *Shigella* (21, 23). Each species offers unique insights into the immune response and their predictive value for human outcomes can vary. Moreover, assessing vaccines across multiple animal models enhances our understanding of their potential in humans. Therefore, to ensure a comprehensive and robust immunological evaluation of altSonflex1-2-3, the three species mentioned previously were selected for this study.

Many studies have indicated association between anti-OAg specific IgG antibodies and protection (41, 42). Other studies have shown that the bactericidal activity of antibodies in sera is associated with reduced clinical disease and may predict vaccine efficacy against *Shigella* infection (43). Therefore, OAg-specific total IgG and levels of functional antibodies were evaluated in this study upon altSonflex1-2–3 immunization.

The immunogenicity of altSonflex1-2–3 was first assessed in preclinical experiments, no negative immunointerference was observed in mice and rabbits, with similar responses against all four GMMA (21). However, the results in terms of immunogenicity obtained in European adult patients, showed a response to *S. flexneri* 1b and 3a GMMA that was numerically lower compared to the one induced by *S. sonnei* and *S. flexneri* 2a GMMA (24). To address these discrepancies and gain a deeper understanding of the vaccine's behavior in animals, we conducted studies using three animal models: mice, rats, and rabbits, testing the vaccine at increasing doses and administered intramuscularly rather than intraperitoneally to more accurately replicate the human route of immunization.

This study aimed to assess the immunogenicity of the altSonflex1-2–3 vaccine across different animal models by evaluating the impact of varying doses and the effect of a second vaccination on the elicited immune response.

## 2 Materials and methods

### 2.1 Strains

*S. sonnei* 53G was obtained from Walter Reed Army Institute of Research, Washington, D.C., USA. The *S. sonnei* Δ*virG*::*cat* strain with stabilized OAg used in FACS and SBA was generated by Caboni et al. (44). *S. flexneri* strains (*S. flexneri* 1b NCTC5, *S. flexneri* 2a NVGH3134, *S. flexneri* 3a NCTC9989) were purchased from Public Health England, London, UK. Frozen 20% glycerol stocks were prepared from lyophilized cultures and stored at −80°C.

### 2.2 Vaccine formulations

altSonflex1-2–3 was produced and characterized according to the methods previously described (21). Briefly, altSonflex1-2–3 was formulated by adsorbing at 1:1:1:1 weight ratio of OAg GMMA from *S. sonnei*, *S. flexneri* serotypes 1b, 2a, and 3a in a solution containing 154 mM NaCl and 10 mM NaH_2_PO_4_ at pH 6.5 and Alhydrogel at a concentration of 0.7 mg/ml (Al^3+^). Further dilutions for immunogenicity studies were performed with Alhydrogel diluent (0.7 mg/ml Al^3+^ in NaCl 154 mM NaH_2_PO_4–_10 mM pH 6.5).

### 2.3 Immunogenicity studies

altSonflex1-2–3 vaccine at different doses was used to immunize mice, rats, and rabbits. GSK is committed to the Replacement, Reduction and Refinement of animal studies (3Rs). Non-animal models and alternative technologies are part of GSK strategy and employed where possible. When animals are required, the application of robust study design principles and peer review minimizes animal use, reduces harm, and improves benefit in studies. Animal studies were ethically reviewed and performed in GSK Animal Resources Center in Siena, Italy (mice and rabbits) and in Charles River Laboratories in Lyon, France (rats) in compliance with relevant guidelines (European Directive 2010/63/UE) and the GSK Policy on the Care, Welfare and Treatment of Animals.

Ten CD1 mice per group (female, 4 to 6 weeks old), eight Sprague Dawley rats (female, 200-300g) per group or six New Zealand white rabbits (female, 2.3-2.7 kg) per group were immunized at day 0 and 28. The number of animals per group was selected to have the 80% power of the study in identifying a at least 4-fold increase in the geometric means of antibody titers between two experimental groups, using the Student's t test and an alpha level of significance of the test equal to 0.05.

All animals were immunized intramuscularly with either 50 µl (mice), 200 µl (rats) or 500 µl (rabbits). Number of doses and injection route reflected those used for European adults enrolled in the clinical trial.

In the Phase 1 clinical trial of altSonflex1-2–3 conducted in European adults, the established human dose involved an injection containing 15 µg of OAg of each serotype, which corresponds to 60 µg of total OAg (24). For preclinical evaluation, varying doses of total OAg were administered to the different animal models. In mice, the tested doses per serotype were 0.06 µg, 0.6 µg, and 6 µg OAg, corresponding to approximately 1/1000th, 1/100th, and 1/10th of the human dose, respectively. Immunization in rats utilized doses of 0.24 µg, 2.4 µg, and 24 µg OAg, which equate to approximately 1/250th, 1/25th, and 1/2.5th of the human dose. In rabbits, the administered doses were 0.6 µg, 6 µg, and 60 µg OAg, representing approximately 1/100th, 1/10th, and 1-fold the human dose, respectively (**Table 1**). To achieve antigen content equivalence to the human dose, the doses were chosen by considering each species' body weight and the practical limitations of the maximum injectable vaccine volume for each species.

**Table 1:** Comparative dosing of altSonflex1-2–3 across preclinical animal models.

Model/Species	Dose (µg of total OAg)	Dose (µg of OAg per serotype)	Relative dose (vs. human dose)
Human (European adults)	60	15	N/A (Reference dose)
Mice	0.06	0.0015	1/1000
	0.6	0.015	1/100
	6	0.15	1/10
Rats	0.24	0.06	1/250
	2.4	0.6	1/25
	24	6	1/2.5
Rabbits	0.6	0.015	1/100
	6	0.15	1/10
	60	15	1/1

The human reference dose is presented alongside scaled doses used for preclinical evaluation in mice, rats, and rabbits, expressed as total OAg amount, OAg amount per serotype and relative to the total human dose. OAg, O Antigen.

Serum was collected on days -1 (baseline), 27 (post-dose 1) and 42 (post-dose 2) and tested in immunoassays.

### 2.4 Enzyme-linked immunosorbent assay

Sera from animals were analyzed using ELISA plates coated with the following antigens: *S. sonnei* lipopolysaccharide (LPS) at 0.5 μg/ml in phosphate-buffered saline (PBS), *S. flexneri* 1b OAg at 2 μg/ml in carbonate buffer, *S. flexneri* 2a OAg at 5 μg/ml in carbonate buffer, and *S. flexneri* 3a OAg at 1 μg/ml in PBS. The plates were then blocked with 5% PBS milk for 1 hour at 25 °C and afterwards incubated with sera diluted at 1:100, 1:4,000, and 1:160,000 in PBS-Tween 0.05% with 0.1% bovine serum albumin (BSA) for mice and rat sera and in 5% milk in PBS for rabbit sera. Following this, IgG detection was conducted using an enzyme-labeled secondary antibody (anti-species specific IgG-alkaline phosphatase, Sigma) in PBS-Tween 0.05% with 0.1% BSA at different concentrations depending on the antigen used and for mice, rabbits, and rats, respectively. The presence of anti-*S. sonnei* LPS/*S. flexneri* 1b, 2a, 3a OAg antibodies was identified by adding a substrate solution, resulting in a yellow color, which was measured by absorbance at 405 nm minus the absorbance at 490 nm. The samples were compared to calibrated species-specific reference standard sera, with results expressed in ELISA units/mL relative to the reference serum. One ELISA unit is defined as the reciprocal of the dilution of the reference serum that produces an OD of one in the assay.

### 2.5 Luminescence based serum bactericidal assay

Serum samples were evaluated for bactericidal activity against altSonflex1-2–3 *Shigella* serotypes by adapting previously published conditions (45, 46). All tested samples were heat inactivated (HI) prior to testing in L-SBA at 56°C for 30 min to remove endogenous complement activity. In brief, bacterial strains were cultured to log-phase (OD_600_ = 0.25 ± 0.02) and then diluted 1:1,000 in PBS. Heat-inactivated sera were diluted in PBS when tested against *S. sonnei* and *S. flexneri* 2a, and 3a strains and in LB when tested against *S. flexneri* 1b strain. Serial dilutions of HI sera from mice, rats, rabbits were added to 96-well plates, followed by the addition of Baby Rabbit Complement (BRC, Cederlane). The concentration of BRC utilized in each assay was different depending on the strain utilized: 30% for *S. flexneri* 1b, 20% for *S. sonnei* and *S. flexneri* 3a, 7.5% for *S. flexneri* 2a. The plates were incubated at 37°C for 3 hours. The viability of the surviving bacteria was assessed using BacTiter-Glo reagent (Promega) to measure ATP production, with luminescence detected via a Synergy HT luminometer (Biotek). The bactericidal activity was quantified by determining the IC50 value, which is defined as the reciprocal serum dilution that leads to a 50% reduction in luminescence (indicating 50% inhibition of bacterial growth). For titers below the minimum measurable signal, a value equal to half of the lowest dilution tested was assigned. Curve fitting and IC50 calculations were performed using GraphPad Prism 9 software.

### 2.6 Statistical analyses

Mann–Whitney two-tailed test was used to compare the total IgG immune response and bactericidal activity elicited by two different groups; Wilcoxon test was used to compare total IgG immune response between post-dose 1 and post-dose 2 for each group.

Dose dependent responses have been evaluated by comparing groups of mice receiving the different doses of GMMA using Spearman rank correlation. The dose response relationship has been considered significant if the P value of the Spearman rank correlation was ≤ 0.05 and the correlation coefficient (Spearman r) was > 0.

Within-animal geometric mean ratios (GMRs) over the baseline values were also calculated for each animal group.

Pearson correlation was used to calculate r coefficients of the correlation between log-transformed total IgG values, measured by ELISA, and log-transformed IC50 values, measured by SBA, in the three animal models.

## 3 Results

### 3.1 Immunogenicity induced by altSonflex1-2–3 in different animal models

#### 3.1.1 Effect of two immunizations of altSonflex1-2–3 at different doses in mice

**Figure 1:**
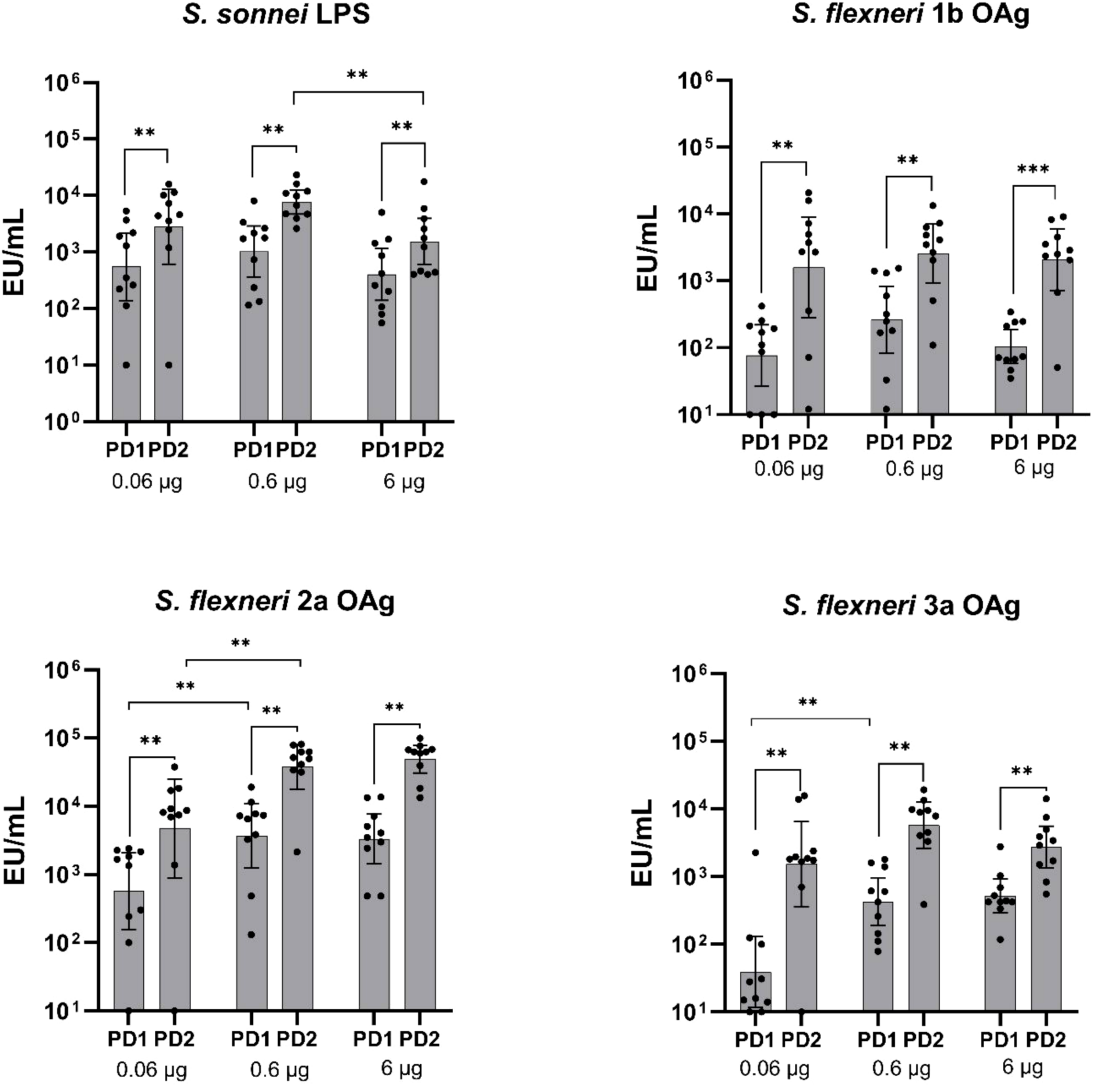
OAg-specific IgG immune response (EU/mL). Groups of 10 mice were immunized IM at Days 0 and 28, and sera were analyzed by ELISA at Day 27 (post-dose 1: PD1) and at Day 42 (post-dose 2 = PD2) in mice vaccinated with three different doses of altSonflex1-2-3 (0.06, 0.6 or 6 µg of total OAg). Geometric mean (bars) and 95% CI (error bars) are reported for all groups together with individual values (dots). The Mann–Whitney test was used for assessing statistical differences between groups and the Wilcoxon test was used for assessing statistical differences between PD1 and PD2 results for each group (**P ≤ 0.01).

When tested in mice, in the range of OAg doses between 0.06–6 mg, altSonflex1-2–3 elicited significant anti-OAg-specific IgG response already post-dose 1, with further increase post-dose 2. Specifically, for all the tested OAg, a significant increase in IgG was observed post-dose 2 compared to post-dose 1 across all three antigen concentrations (P ≤ 0.01, **; **Figure 1**).

Spearman rank correlation results to verify dose-dependent responses are summarized in **Table 2**. 27 days after first injection, a significant correlation was found for *S. flexneri* 2a OAg (Spearman's rank correlation 0.4860, P ≤ 0.01, **) and *S. flexneri* 3a OAg (Spearman's rank correlation 0.5691, P ≤ 0.01, **), but not for *S. sonnei* and *S. flexneri* 1b OAg (**Table 2**). Post-dose 2, a significant increase in IgG at the increase of the total OAg dose in the vaccine formulation was observed only for *S. flexneri* 2a (Spearman's rank correlation 0.6650, P ≤ 0.0001, ****). Notably, for *S. sonnei*, the 6 µg dose of total OAg elicited significantly lower IgG compared to the 0.6 µg dose (P ≤ 0.01, **) (**Table 2**).

**Table 2:** Evaluation of the OAg-specific IgG immune response and serum bactericidal activity at three different altSonflex1-2–3 doses in mice, rats, and rabbits.

Immune response		Mice			Rats			Rabbits		
Dose (µg total OAg)		0.06	0.6	6	0.24	2.4	24	0.6	6	60
*Increase of IgG response after boost (PD2) ^a^*	Sso	+	+	–	+	+	–	–	–	–
	Sf1b	+	+	+	+	+	+	+	+	–
	Sf2a	+	+	+	+	+	+	+	+	–
	Sf3a	+	+	+	+	+	–	+	+	+
*IgG dose-response correlation PD1 ^b^*	Sso	-0.169 (0.369)			-0.088 (0.681)			0.384 (0.127)		
	Sf1b	0.018 (0.921)			0.700 (0.000)			0.391 (0.120)		
	Sf2a	0.486 (0.006)			0.52 (0.008)			0.657 (0.005)		
	Sf3a	0.569 (0.001)			0.287 (0.173)			0.700 (0.002)		
*IgG dose-response correlation PD2 ^c^*	Sso	-0.259 (0.166)			-0.081 (0.706)			-0.050 (0.846)		
	Sf1b	-0.070 (0.710)			-0.154 (0.470)			0.478 (0.054)		
	Sf2a	0.665 (0.000)			0.449 (0.027)			0.435 (0.082)		
	Sf3a	0.070 (0.710)			-0.184 (0.388)			0.726 (0.001)		
*Serum bactericidal activity dose-response correlation PD2 ^d^*	Sso	0.042 (0.823)			-0.007 (0.972)			0.193 (0.452)		
	Sf1b	0.084 (0.655)			-0.147 (0.491)			0.708 (0.002)		
	Sf2a	0.669 (0.000)			0.3907 (0.059)			0.584 (0.015)		
	Sf3a	-0.023 (0.901)			0.051 (0.810)			0.757 (0.000)		

(a) boost effect observed after the second dose (PD2) for antigen-specific IgG: “+” indicates a boost effect, “–” indicates no boost effect. (b) Spearman correlation between dose and IgG response at PD1, (c) Spearman correlation between dose and IgG response at PD2, (d) Spearman correlation between dose and serum bactericidal activity at PD2. For panels (b), (c), and (d), values are expressed as Spearman r (P value). ns = not significant; Sso = *S. sonnei*; Sf1b = *S. flexneri* 1b; Sf2a = *S. flexneri* 2a; Sf3a = *S. flexneri* 3a.

**Figure 2:**
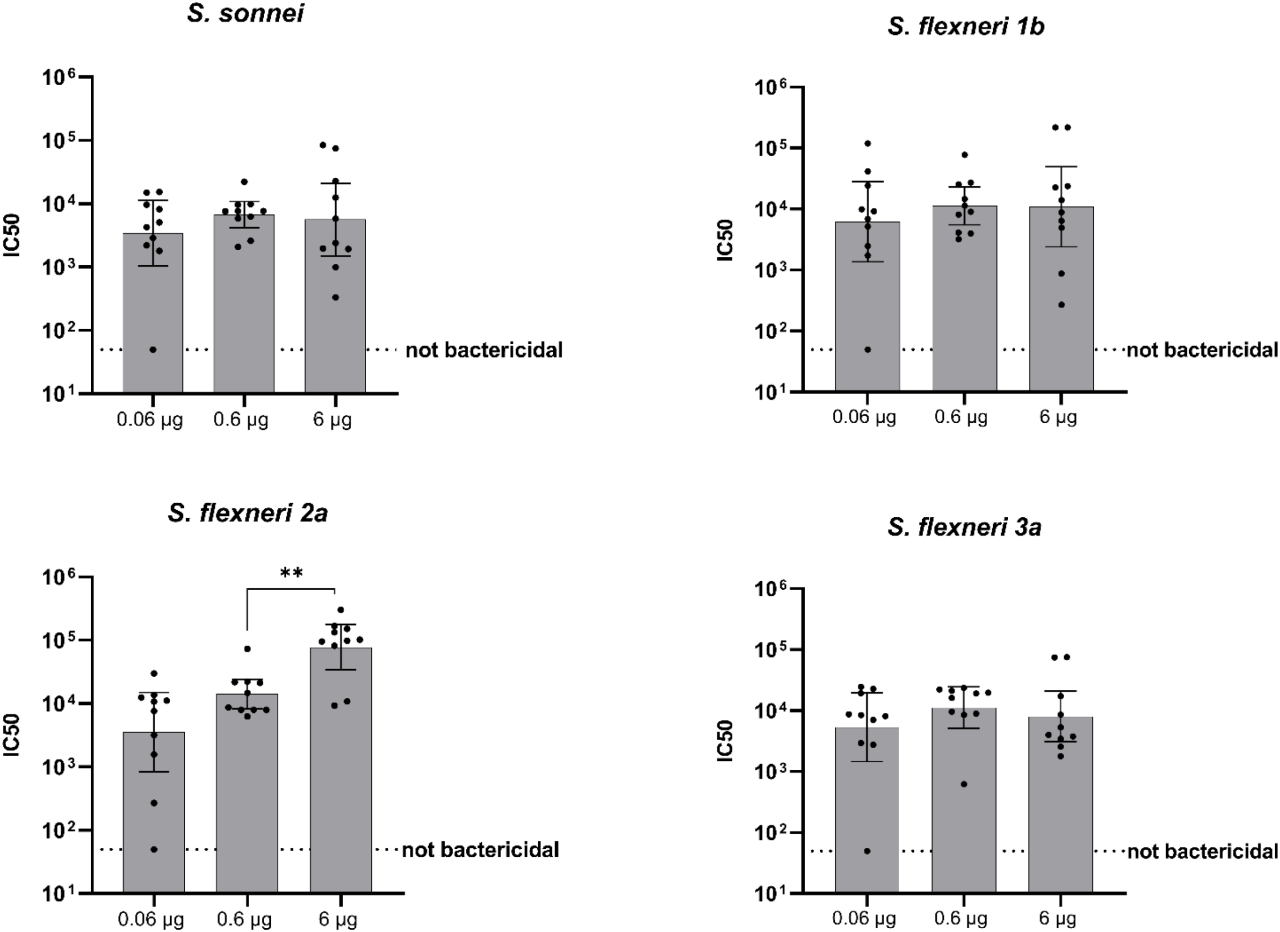
Bactericidal activity of antibodies (IC50). Groups of 10 mice were immunized IM at days 0 and 28, and sera were analyzed by SBA at Day 42 (post-dose 2) in mice vaccinated with three different doses of altSonflex1-2-3 (0.06, 0.6 or 6 µg of total OAg). Bactericidal activity was determined as the dilution necessary to obtain 50% CFU reduction at T180 compared with T0. Geometric mean (bars) and 95% CI (error bars) are reported for all groups together with individual values (dots). Baseline values (Day -1) are indicated by the dotted line. The Mann–Whitney test was used for assessing statistical differences between groups (**P ≤ 0.01).

Sera collected post-dose 2 were also assessed for serum bactericidal activity. No dose response was observed, except for *S. flexneri* 2a, for which a positive correlation between the increase of the OAg dose and bactericidal activity was observed (Spearman's rank correlation 0.6698, P ≤ 0.0001, ****) (**Figure 2**, **Table 2**).

#### 3.1.2 Effect of two immunizations of altSonflex1-2–3 at different doses in rats

**Figure 3:**
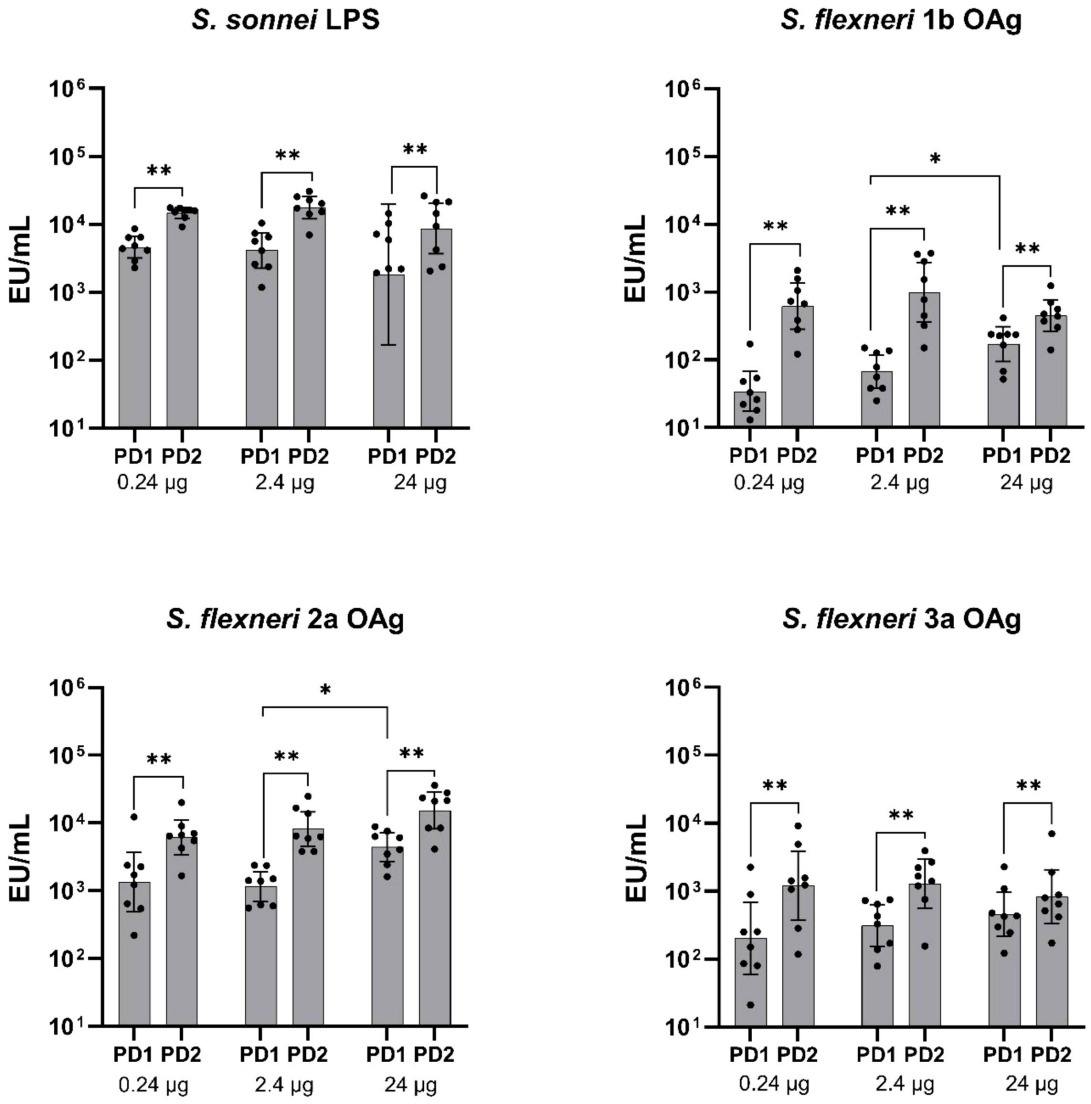
OAg-specific IgG immune response (EU/mL). Groups of eight rats were immunized IM at days 0 and 28, and sera were analyzed by ELISA at Day 27 (post-dose 1: PD1) and at Day 42 (post-dose 2 = PD2) in rats vaccinated with three different doses of altSonflex1-2-3 (0.24, 2.4 or 24 µg of total OAg). Geometric mean (bars) and 95% CI (error bars) are reported for all groups together with individual values (dots). The Mann–Whitney test was used for assessing statistical differences between groups and the Wilcoxon test was used for assessing statistical differences between PD1 and PD2 results for each group (*P ≤ 0.05, **P ≤ 0.01).

When tested in rats, in the range of OAg doses between 0.24-24 μg, altSonflex1-2–3 elicited a strong immune response in terms of OAg-specific IgG against all the four antigens, already after the first immunization. IgG levels further increased post-dose 2 (**Figure 3**). In particular, a significant increase in OAg-specific IgG post-dose 2 was observed at all the antigen doses tested against all four *S. sonnei* and *S. flexneri* OAg (P ≤ 0.01, **).

A dose-dependent OAg-specific total IgG response was observed post-dose 1 against *S. flexneri* 1b OAg (Spearman's rank correlation 0.7007, P ≤ 0.001, ***) and *S. flexneri* 2a OAg (Spearman's rank correlation 0.5234, P ≤ 0.01, **), but not against *S. sonnei* and *S. flexneri* 3a OAg (**Table 2**). Following the second vaccination, a significant positive correlation between total OAg dose and IgG levels was found exclusively for *S. flexneri* 2a (Spearman's rank correlation 0.4497, P ≤ 0.05, *).

**Figure 4:**
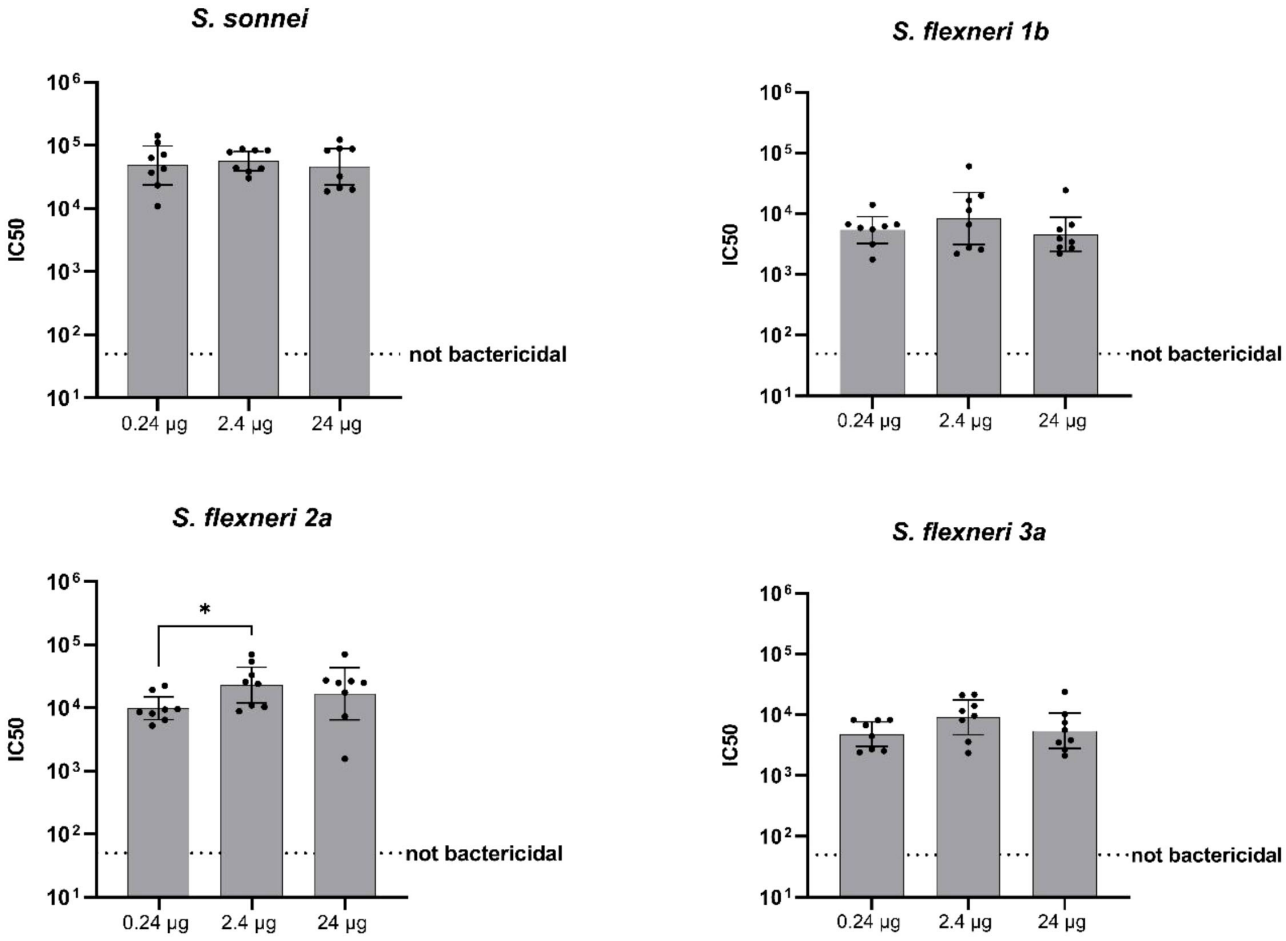
Bactericidal activity of antibodies (IC50). Groups of eight rats were immunized IM at days 0 and 28, and sera were analyzed by SBA at Day 42 (post-dose 2) in rats vaccinated with three different doses of altSonflex1-2-3 (0.24, 2.4 or 24 µg of total OAg). Bactericidal activity was determined as the dilution necessary to obtain 50% CFU reduction at T180 compared with T0. Geometric mean (bars) and 95% CI (error bars) are reported for all groups together with individual values (dots). Baseline values (Day -1) are indicated by the dotted line. The Mann–Whitney test was used for assessing statistical differences between groups (*P ≤ 0.05).

For the SBA titers post dose 2, instead, no dose-response correlation was found against any of the four strains evaluated (**Figure 4**, **Table 2**).

#### 3.1.3 Effect of two immunizations of altSonflex1-2–3 at different doses in rabbits

**Figure 5:**
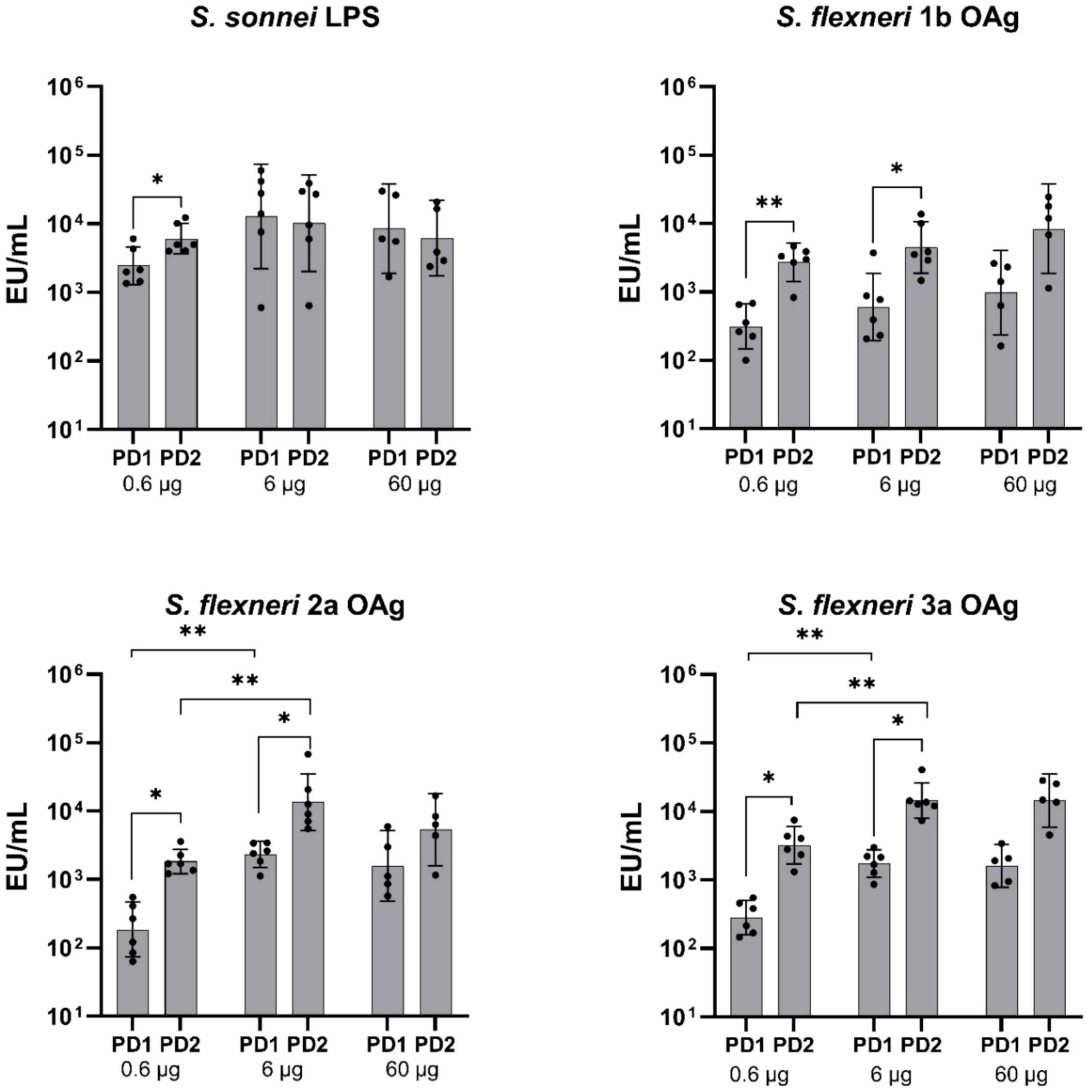
OAg-specific IgG immune response (EU/mL). Groups of six rabbits were immunized IM at days 0 and 28, and sera were analyzed by ELISA at Day 27 (post-dose 1: PD1) and at Day 42 (post-dose 2 = PD2) in rabbits vaccinated with three different doses of altSonflex1-2-3 (0.6, 6 or 60 µg of total OAg). Geometric mean (bars) and 95% CI (error bars) are reported for all groups together with individual values (dots). The Mann–Whitney test was used for assessing statistical differences between groups and the Wilcoxon test was used for assessing statistical differences between PD1 and PD2results for each group (*P ≤ 0.05, **P ≤ 0.01).

In rabbits, in the range of OAg doses tested between 0.6–60 μg, the first immunization elicited a robust IgG response against all four target antigens (**Figure 5**). A booster effect was observed following the second dose, with IgG titers significantly increasing for all the OAg but not at all the three antigen concentrations assessed. Specifically, for *S. flexneri* 1b OAg, a significant elevation in IgG levels post-dose 2 was achieved for the 0.6 µg (P ≤ 0.01, **) and 6 µg (P ≤ 0.05, *) doses. A similar pattern was noted for *S. flexneri* 2a and 3a OAg, where both the 0.6 µg and 6 µg dose resulted in a significant increase (P ≤ 0.05, *), but not the 60 µg of total OAg dose. IgG response specific to *S. sonnei* OAg significantly increased post-dose 2 at the lower total OAg concentration tested of 0.6 µg (P ≤ 0.05, *), while for the two higher concentrations of 6 and 60 µg of total OAg dose, IgG titers remained comparable to the response observed post-dose 1.

Post-dose 1, a dose-dependent total IgG response was observed only against *S. flexneri* 2a OAg, with a Spearman's rank correlation of 0.6574 (P ≤ 0.001, **), and against *S. flexneri* 3a OAg with a correlation of 0.7003 (P ≤ 0.01,**). However, no dose response was observed for *S. sonnei* and *S. flexneri* 1b OAg (**Table 2**). Following the second vaccination, a significant positive correlation between the total OAg dose and IgG levels was detected exclusively for *S. flexneri* 3a (Spearman's rank correlation 0.7263, P ≤ 0.01,**).

**Figure 6:**
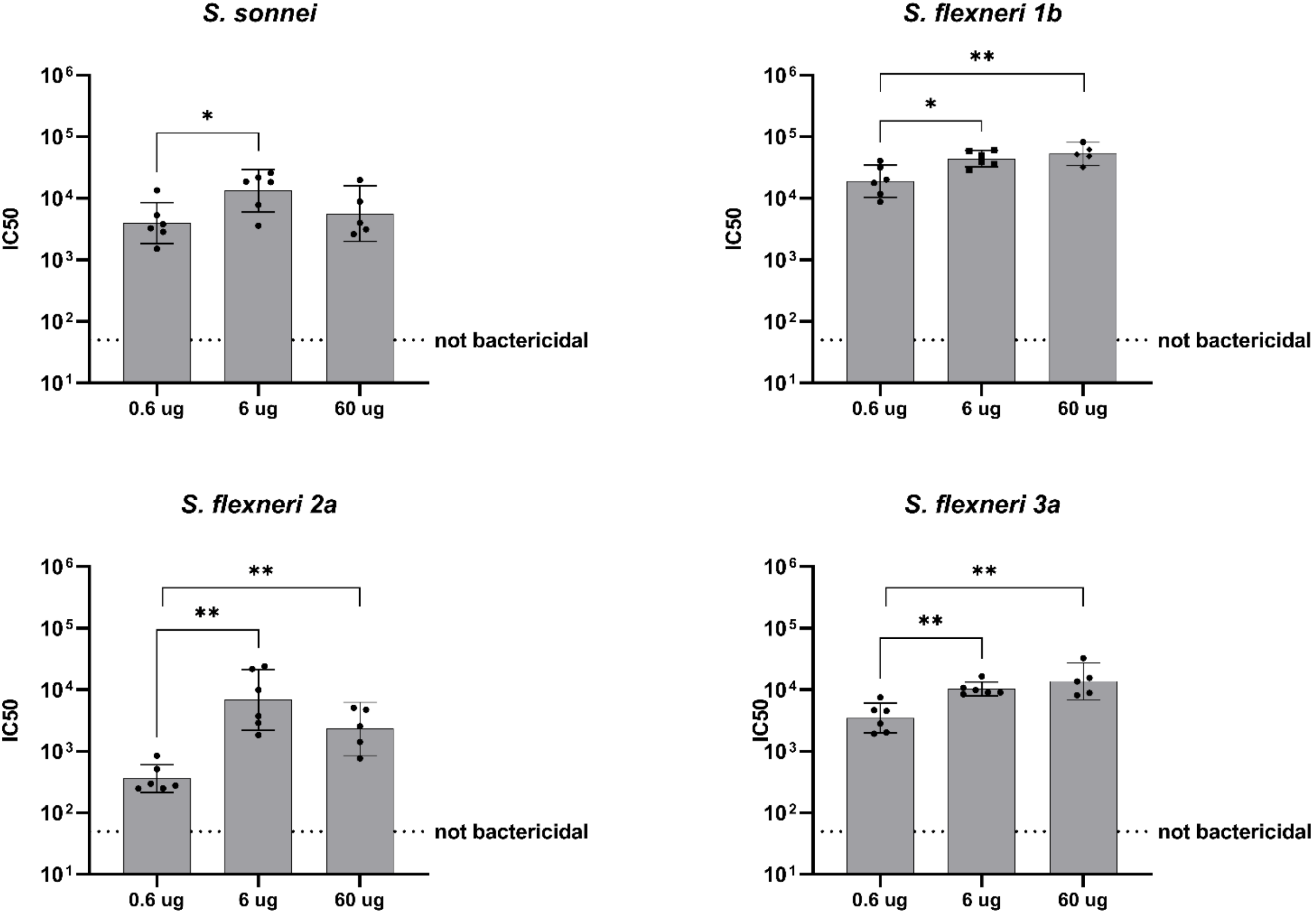
Bactericidal activity of antibodies (IC50). Groups of six rabbits were immunized IM at days 0 and 28, and sera were analyzed by SBA at Day 42 (post-dose 2) in rabbits vaccinated with three different doses of altSonflex1-2-3 (0.6, 6 or 60 µg of total OAg). Bactericidal activity was determined as the dilution necessary to obtain 50% CFU reduction at T180 compared with T0. Geometric mean (bars) and 95% CI (error bars) are reported for all groups together with individual values (dots). Baseline values (Day -1) are indicated by the dotted line. The Mann–Whitney test was used for assessing statistical differences between groups (* P ≤ 0.05, ** P ≤ 0.01).

In terms of SBA, a dose-response correlation was evident against the three *S. flexneri* strains tested. In particular, bactericidal activity increase correlated with dose increase in the case of SBA against *S. flexneri* 1b (Spearman's rank correlation 0.7081, P ≤ 0.01, **), *S. flexneri* 2a (Spearman's rank correlation 0.5847, P ≤ 0.05, *) and *S. flexneri* 3a (Spearman's rank correlation 0.7575, P ≤ 0.001, ***) (**Figure 6**, **Table 2**).

#### 3.1.4 Relative comparison of the immune response induced by the different components of altSonflex1-2-3

**Figure 7:**
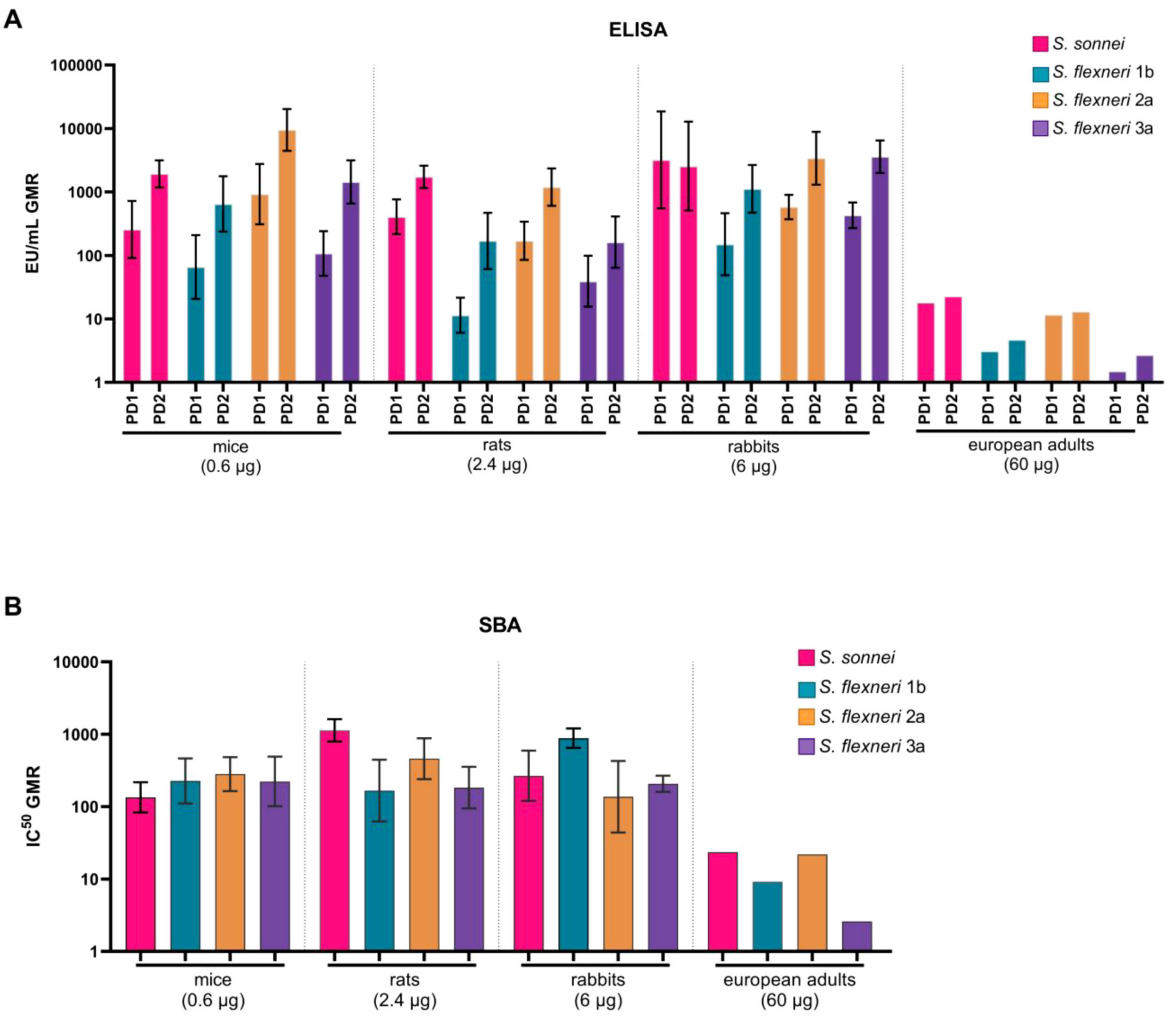
Comparison of humoral immune responses after vaccination with altSonflex1-2–3 between mice (0.6 µg total OAg dose), rats (2.4 µg total OAg dose), rabbits (6 µg total OAg dose) and European adults (60 ug total OAg dose) in terms of: **(A)***S. sonnei* and *S. flexneri* 1b, 2a and 3a OAg-specific IgG post-dose 1 (PD1) and post-dose 2 (PD2). Geometric mean ratio over the baseline values (bars) for all the groups and 95% CI (error bars) for the preclinical studies groups are reported; **(B)** IC^50^ of bactericidal activity against *S. sonnei* and *S. flexneri* 1b, 2a and 3a strains. Geometric mean ratio over the baseline values (bars) for all the groups and 95% CI (error bars) for the preclinical studies groups are reported.

In order to compare the immune response elicited by the four components of the vaccine, antigen-specific IgG and SBA responses were reported as GMR over baseline. In mice administered with 0.6 μg of total OAg, the ELISA results showed a largely homogeneous IgG response across the four antigens, with the notable exception of *S. flexneri* 2a, which exhibited particularly high GMRs (925.8 at PD1 and 9501 at PD2) compared with *S. flexneri* 1b (65.8 PD1 and 647.2 PD2), *S. flexneri* 3a (107.4 PD1 and 1445.3 PD2), and *S. sonnei* (257.2 PD1 and 1929.8 PD2). Corresponding SBA values in mice followed a similar trend, with GMRs of 134.6 for *S. sonnei*, 226.6 for *S. flexneri* 1b, 282 for *S. flexneri* 2a, and 223.1 for *S. flexneri* 3a (**Figure 7**). In rats, ELISA measurements indicated higher IgG GMRs for *S. sonnei* OAg (407.5 at PD1 and 1737.3 at PD2) and *S. flexneri* 2a OAg (170.4 at PD1 and 1192.5 at PD2) compared with those for *S. flexneri* 1b (11.4 PD1 and 169.6 PD2) and *S. flexneri* 3a (39.3 PD1 and 161.8 PD2). The SBA results in rats paralleled these findings, with GMRs of 1134.9 for *S. sonnei* and 460.1 for *S. flexneri* 2a, whereas values for *S. flexneri* 1b and *S. flexneri* 3a were lower (166.9 and 183.2, respectively). This pattern in rats mirrors closely the trends observed in European adults (24). In rabbits, the pattern of responses differed. ELISA data indicated that PD2, IgG titers were comparable for three antigens, *S. sonnei* OAg (GMR of 2553), *S. flexneri* 2a OAg (GMR of 3397.8), and *S. flexneri* 3a OAg (GMR of 3607.9), with a relatively lower response against *S. flexneri* 1b OAg (GMR of 1123). However, the SBA results in rabbits did not confirm the ELISA findings in terms of homogeneity of the immune response among the four vaccine strains, as bactericidal GMRs for *S. flexneri* 1b (GMR of 883.1) were higher than those for *S. sonnei* (GMR of 266.6), *S. flexneri* 2a (GMR of 137.1), and *S. flexneri* 3a (GMR of 206.9).

To facilitate the comparison with data from European adults immunized with altSonflex1-2–3 at a dose corresponding to 60 μg total OAg, the intermediate dose used in each of the animal models was selected (**Figure 7**). Data from the higher and lower doses used in the preclinical settings are shown in the **Supplementary Material (Supplementary Figures 1, 2)** to provide further insights into the dose-dependent effects in the three animal models of altSonflex1-2-3.

Interestingly, the overall analysis of the results obtained in the three species showed that SBA titres correlated differently based on the different species with the total IgG response (**Table 3**). In particular, in mice a strong correlation was found between anti-*S. flexneri* 3a OAg IgG response and bactericidal activity (Pearson r = 0.74, P ≤ 0.001) and moderate correlation between *S. flexneri* 2a (Pearson r = 0.50, P ≤ 0.01) OAg IgG responses and bactericidal activity. A weaker, albeit significant, correlation was observed for anti-*S. sonnei* OAg IgG (Pearson r = 0.37, P ≤ 0.05) and no significant correlation was found for *S. flexneri* 1b. For what concerns the correlation in rats, only a moderate correlation between IgG responses and bactericidal activity against *S. sonnei* (Pearson r = 0.53, P ≤ 0.01) was observed, but weak correlation against *S. flexneri* 1b (Pearson r = 0.48, P ≤ 0.05) or no significant correlation was found for *S. flexneri* 2a and 3a. In the rabbit model, a moderate correlation was found between IgG responses and bactericidal activity against *S. sonnei* (Pearson r = 0.56, P ≤ 0.05), and strong correlations against *S. flexneri* 1b (Pearson r = 0.73, P ≤ 0.001), *S. flexneri* 2a (Pearson r = 0.91, P ≤ 0.001), and *S. flexneri* 3a (Pearson r = 0.93, P ≤ 0.001).

**Table 3:** Pearson correlation coefficients (Pearson r values) and p-values of the correlation among *S. sonnei*, *S. flexneri* 1b, 2a and 3a OAg-specific IgG responses and bactericidal activity in mice, rats, and rabbits. The OAg-specific IgG response detected in all mice, rats and rabbits immunized with three different doses of altSonflex1-2–3 was correlated with serum bactericidal activity titers. Values close to 1 or –1 indicate a strong correlation while values close to 0 indicate a low correlation. n = 30 for mice, 24 for rats, 18 for rabbits.

Pearson correlation coefficients of IgG response vs. bactericidal activity (and p-values of the correlation)			
	Mice	Rats	Rabbits
*S. sonnei*	0.37 (0.045)	0.53 (0.008)	0.56 (0.020)
*S. flexneri* 1b	0.34 (0.069)	0.48 (0.017)	0.73 (0.001)
*S. flexneri* 2a	0.74 (0.000)	-0.05 (0.835)	0.91 (0.000)
*S. flexneri* 3a	0.50 (0.005)	0.36 (0.082)	0.93 (0.000)

## 4 Discussion

Shigellosis remains one of the leading causes of diarrheal disease in low- and middle-income countries, particularly affecting young children (2). The growing concern over antimicrobial resistance in *Shigella* further exacerbates the public health challenge. Currently, no licensed vaccines exist against *Shigella*, though several candidates that target the OAg portion of lipopolysaccharides are in development (47). GVGH proposed GMMA as an innovative delivery system for the OAg (48) leading to the development of a four-component vaccine candidate, altSonflex1-2-3. This vaccine includes GMMA from *S. sonnei* and three prevalent and epidemiologically relevant *S. flexneri* serotypes (1b, 2a, and 3a) and is currently undergoing Phase 2 clinical trials (21, 24).

In this study, we compared four altSonflex1-2–3 in three different animal models (mouse, rat, and rabbit) for their ability to (i) boost immune responses after a second dose, (ii) elicit proportional IgG functional responses to the total OAg dose administered, and (iii) induce comparable immune responses across the four OAg components.

Different behaviors were observed among the three animal species and compared to humans in terms of boosting of the immune response after a second vaccination. In mice, there was a significant increase in IgG titers against all four OAg delivered by the vaccine after the second dose (**Figure 1**). Rats exhibited a similar response, with a noticeable increase in IgG levels post-dose 2 (**Figure 3**). Conversely, in rabbits, a boost effect was evident for *S. flexneri* OAg only at the lower and intermediate total OAg doses tested, and there was no significant change in IgG response against *S. sonnei* OAg between the first and second vaccination, except for the lower total OAg dose tested (**Figure 5**), possibly due to antigen overload causing immune system saturation. In humans, although there was a slight numerical increase in IgG levels between post-dose 1 and post-dose 2, initial immunogenicity results from healthy European adults indicated that altSonflex1-2–3 successfully elicited OAg-specific total IgG levels and bactericidal titers against the four vaccine components after a single immunization, and that the second immunization restored the immune response reached after the first one (24). Despite in animals a more pronounced boost effect was observed in most of the cases respect to what observed in humans, it is important to emphasize that a single immunization with altSonflex1-2–3 consistently induced already a strong primary immune response in all tested systems.

To evaluate the effect of different antigen doses, OAg-specific IgG and functional antibody activity were measured in animals following administration of three increasing doses of the vaccine (scaled by animal weight relative to the full human dose). Overall, the results indicate that significant dose-response correlations were antigen/model dependent. For example, in mice a significant dose-response was observed post-dose 1 for IgG against *S. flexneri* 2a and 3a OAg, with a corresponding trend in bactericidal activity against *S. flexneri* 2a OAg (**Table 2**). In rats, significant dose-response increases were recorded for *S. flexneri* 1b and 2a OAg post-dose 1, whereas after the second immunization a dose-response remained only for *S. flexneri* 2a and SBA data did not reveal consistent trends (**Table 2**). In rabbits, dose-response relationships were more apparent: OAg-specific IgG correlated with dose for *S. flexneri* 2a and 3a OAg after the first dose, although after the second dose the effect persisted only for *S. flexneri* 3a. SBA responses in rabbits showed a clear dose-dependent increase against all three *S. flexneri* strains (**Table 2**). Overall, although not uniformly observed, these findings highlight species- and antigen-specific dose dependencies, with *S. flexneri* 2a emerging as the antigen most consistently showing a dose-response relationship across models. Importantly, across all three animal models the highest vaccine dose administered occasionally resulted in reduced OAg-specific IgG, most markedly against *S. sonnei* OAg, suggesting a ‘’hook effect’’. The observed ‘’hook effect’’ indicates that very high altSonflex1-2–3 dose can lead to suboptimal immunological responses to vaccination and this is an important aspect to be considered while planning clinical studies. Indeed, in the altSonflex1-2–3 Phase 2 trials, three different total OAg doses are being evaluated in infants, the target population. This will allow the comparison of preclinical results presented here with the immune responses observed in infants, thereby determining whether the dose-response behavior observed in animal models is recapitulated in humans. Previous insights from clinical studies testing the *S. sonnei* 1790GAHB monocomponent vaccine in European adults in the range 0.059-5.9 μg OAg dose showed that there was a significant correlation between dose and antibody response on day 85, i.e. 28 days post 3^rd^ vaccination (Spearman rank ρ = 0.529, P = 0.00013). The response peaked with the 1.5 μg OAg dose, and the antibody responses to the 1.5, 2.9 and 5.9 μg doses were not significantly different (49). In African adults, antibody levels increased already post-first vaccination: 2.10- and 4.43-fold from baseline values in the 1.5 and 5.9 OAg μg groups, respectively, and no significant increase was observed after second injection (50). All the analyses presented in this work were conducted using two principal immunological readouts: OAg-specific IgG measured by ELISA and functional activity measured by SBA. These same readouts have been adopted in the Phase 1 trial in European adults as the primary criteria to evaluate immunogenicity (24), and will also serve as the primary endpoints in the ongoing Phase 2 trials. Previous studies have demonstrated a correlation between anti-*S. sonnei* OAg IgG levels and bactericidal activity in subjects vaccinated with the *S. sonnei* (1790GAHB) monocomponent candidate vaccine (51). Similar findings were observed in mice immunized with the same candidate, where bactericidal activity was predominantly associated with the IgG1 and IgG3 subclasses (52). In the current study, we extended these observations by examining not only the response to *S. sonnei* but also to *S. flexneri* 1b, 2a, and 3a serotypes in the 4-component formulation. Furthermore, this analysis spanned three different animal models (mouse, rat, and rabbit) using a range of different doses of altSonflex1-2-3. Results obtained in this work show that while mice exhibit moderate correlations for certain serotypes, rabbits consistently demonstrate strong correlations between IgG levels and bactericidal activity across multiple serotypes. Rats, however, display less consistent associations, highlighting also in this case clear species-dependent differences. Interestingly, in European adults who received altSonflex1-2–3 at a dose of 60 µg of total OAg, the results indicate a less pronounced association between OAg-specific IgG levels and bactericidal activity (24) compared to observations in mouse and rabbit models. In contrast, the responses observed in rats align more closely with the Phase 1 outcomes, further emphasizing the potential translational relevance of the rat model for this vaccine. The correlation between OAg-specific responses to the altSonflex1-2–3 vaccine antigens and bactericidal activity will be also investigated in subjects enrolled in the Phase 2 trial, this analysis will further help to determine which of the tested animal models best reflects the correlation observed in the vaccine target population.

Finally, in this work the homogeneity of the immune response among the four antigens was evaluated in the three animal models and the results were compared with clinical results from European adults from Phase 1 clinical trial. Indeed, from the Phase1 clinical trial in which subjects were vaccinated with altSonflex1-2–3 at a dose of 60 µg of total OAg, a greater immune response was observed both in terms of OAg-specific IgG titers and bactericidal activity of the serum antibodies against *S. sonnei* and *S. flexneri* 2a, compared to *S. flexneri* 1b and *S. flexneri* 3a (24). Among animals, data revealed that the homogeneity of the antigen-specific responses varied among species. The rat model most closely reflected the clinical pattern with higher responses to *S. sonnei* and *S. flexneri* 2a, both post first and second vaccination (**Figure 7**). Indeed, rats have been extensively utilized in vaccine research to study immune responses (53–56). **Supplementary Figures 1, 2** indicate that the overall homogeneity of the response to the four antigens is also maintained at different doses of total OAg administered in each of the three animal models. The observed variations across the immune response to the four different serotypes may be due to factors such as negative immunointerference, where the high total OAg dose and the corresponding high protein content may have led to carrier suppression, particularly for *S. flexneri* 1b and 3a. This phenomenon was already observed for protein-based and glycoconjugate vaccines (57, 58). It is also important to highlight that some of the discrepancies observed between pre-clinical animal models and clinical human results can also be attributed to baseline differences in IgG titers. Animals exhibit no detectable baseline levels of *Shigella* OAg-specific IgG titers, whereas European adults in our study showed modest baseline titers, particularly against *S. flexneri* 1b and *S. flexneri* 3a (24). Future clinical data in the target population could provide a more comprehensive comparison (24). Indeed, the immunogenic response in infants could represent differences compared to adults due to developmental variations in the immune system across age groups.

In general, the study highlights differences and similarities in vaccine immunogenicity across different species. Differences in the immune responses across mice, rats and rabbits probably derive from species-specific features. For example, differences in the MHC class II genes could lead to different antigen presentation (59). Moreover, rabbits further display unique immunological features that produce antibody responses distinct from rodents (36).

This study represents a rare exploration of the immune response elicited by a vaccine candidate across various animal models. This approach enhances the predictive value of preclinical studies by integrating data from models that represent different immunological and physiological landscapes, ultimately accelerating the development of effective and safe GMMA-based and multivalent bacterial vaccines. Broadening research of this nature to include diverse platforms and pathogens has the potential to yield valuable information about the most suitable animal models to predict human results and for accelerating vaccine design and development ahead of clinical trials.

## Data Availability

The raw data supporting the conclusions of this article will be made available by the authors, without undue reservation.
