# Supplementary material for: Immunogenicity evaluation of altSonflex1-2–3 Shigella vaccine across mice, rats, and rabbits to inform human translational insights
Source: Front Immunol. 2026 Jan 21;16:1740821. doi: 10.3389/fimmu.2025.1740821 (PMC12868232; doi:10.3389/fimmu.2025.1740821)
Supplement: Supplementary file 1 [file SupplementaryFile1.docx]

Supplementary Material


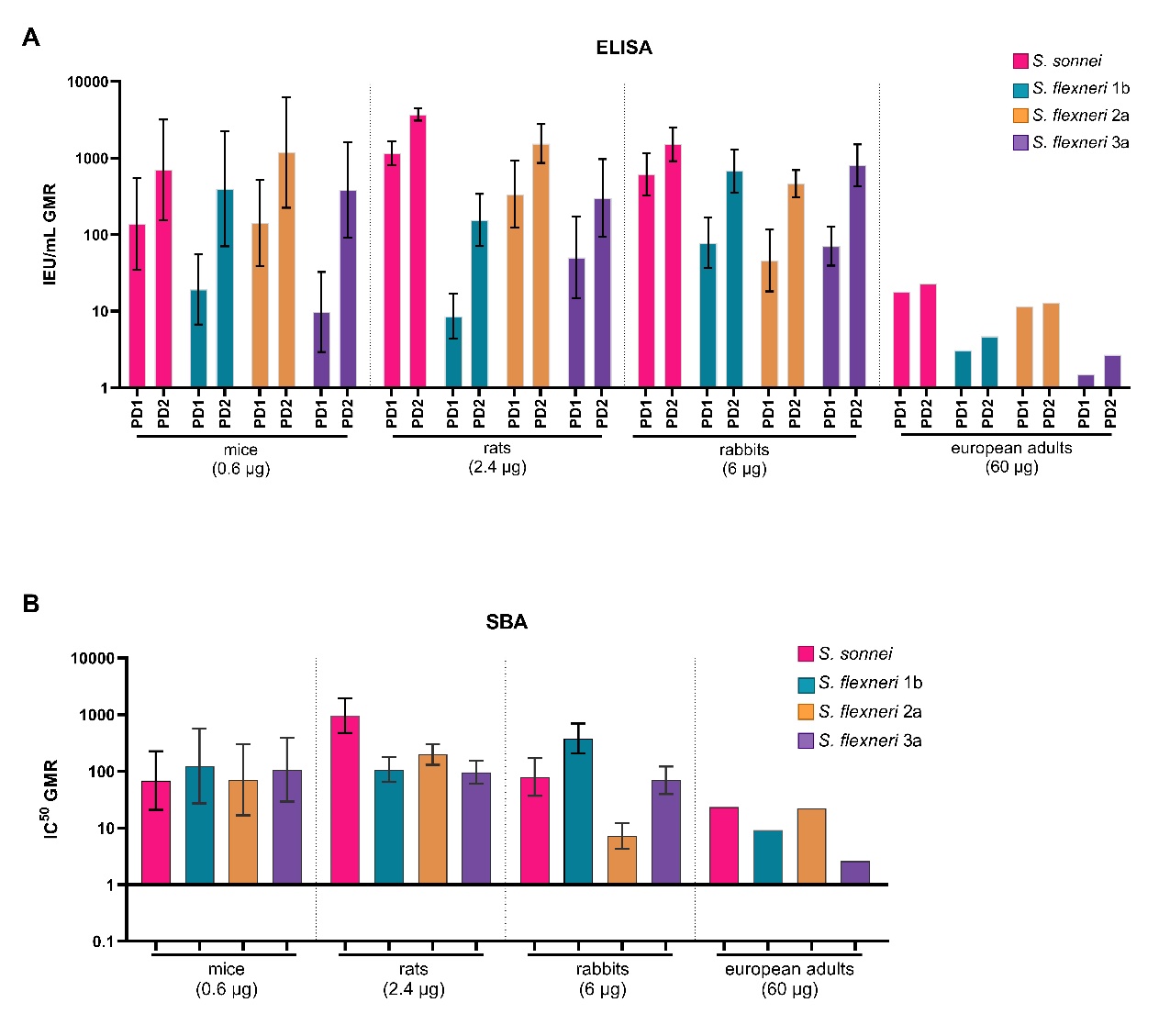


**Supplementary Figure 1.** Comparison of humoral immune responses after vaccination with altSonflex1-2-3 between mice (0.06 µg tot. OAg dose), rats (0.24 µg tot. OAg dose), rabbits (0.6 µg tot. OAg dose), and European adults in terms of: A: *S. sonnei* and *S. flexneri* 1b, 2a and 3a OAg-specific IgG levels post-dose 1 (PD1) and post-dose 2 (PD2). Geometric mean ratio of IgG titers over the baseline values (bars) for all the groups and 95% CI (error bars) for the preclinical studies groups are reported; B: bactericidal activity against *S. sonnei* and *S. flexneri* 1b, 2a and 3a strains post-dose 2. Geometric mean ratio of bactericidal titers over the baseline values (bars) for all the groups and 95% CI (error bars) for the preclinical studies groups are reported.


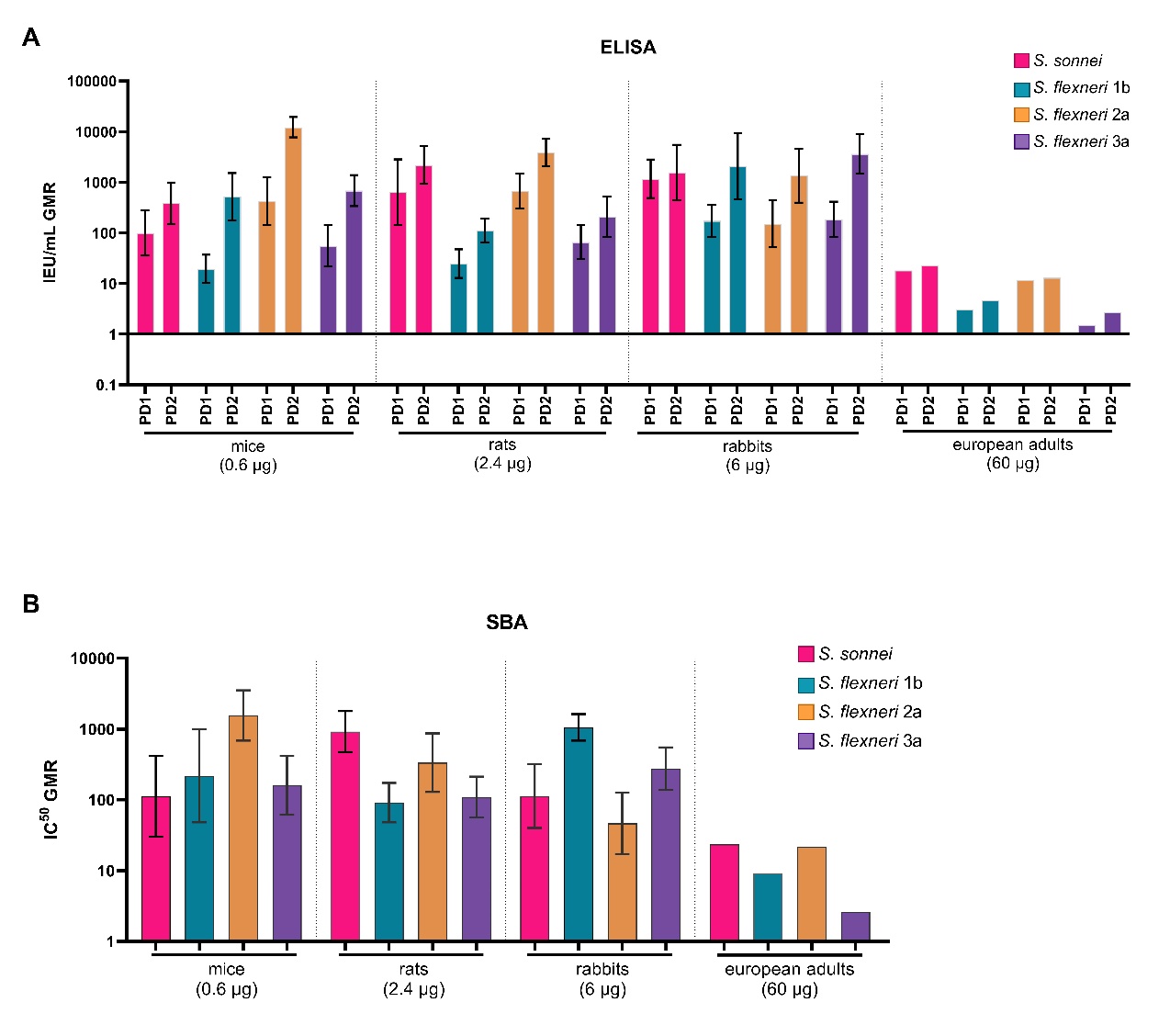


**Supplementary Figure 2.** Comparison of humoral immune responses after vaccination with altSonflex1-2-3 between mice (6 µg tot. OAg dose), rats (24 µg tot. OAg dose), rabbits (60 µg tot. OAg dose), and European adults in terms of: A: *S. sonnei* and *S. flexneri* 1b, 2a and 3a OAg-specific IgG levels post-dose 1 (PD1) and post-dose 2 (PD2). Geometric mean ratio of IgG titers over the baseline values (bars) for all the groups and 95% CI (error bars) for the preclinical studies groups are reported; B: bactericidal activity against *S. sonnei* and *S. flexneri* 1b, 2a and 3a strains post-dose 2. Geometric mean ratio of bactericidal titers over the baseline values (bars) for all the groups and 95% CI (error bars) for the preclinical studies groups are reported.
